# Enhanced catalytic activity of MOF-74 via providing additional open metal sites for cyanosilylation of aldehydes

**DOI:** 10.1038/s41598-022-18932-z

**Published:** 2022-08-30

**Authors:** Hyeji Jun, Sojin Oh, Gihyun Lee, Moonhyun Oh

**Affiliations:** Department of Chemistry, Yonsei University, 50 Yonsei-ro, Seodaemun-gu, Seoul 03722 Republic of Korea

**Keywords:** Metal-organic frameworks, Inorganic chemistry

## Abstract

The preparation of metal-organic frameworks (MOFs) having many open metal sites is an excellent approach for the development of highly active MOF-based catalysts. Herein, well-defined rice-shaped MOF-74 microparticles having structural defects are prepared by incorporating two analogous organic linkers [2,5-dihydroxy-1,4-bezenedicarboxylic acid (DHBDC) and 2-hydroxy-1,4-benzenedicarboxylic acid (HBDC)] within the MOF-74 structure. The replacement of some of DHBDC in MOF-74 by HBDC causes the structural defects (excluding some of the bridged hydroxyl groups), and these structural defects provide the additional open metal sites within MOF-74. Finally, the additional open metal sites within MOF-74 result in the enhanced catalytic activity for the cyanosilylation of several aldehydes. A series of MOF-74s is prepared with various incorporated amounts of HBDC, and the optimum ratio between DHBDC and HBDC in MOF-74 to achieving the best catalytic performance is determined. In addition, the defected MOF-74 displays an excellent recyclability for the catalytic reaction.

## Introduction

Metal−organic frameworks (MOFs) are an important class of microporous and mesoporous crystalline materials that are widely used for many practical applications, such as gas storage, recognition, sensing, and catalysis^1–20^. Generally, MOFs can be constructed from a wide range of organic linkers and metal ions, and their properties are mostly governed by their compositional and structural features^21–27^. Hence, researchers are attempting to tune their compositions and structures to achieve an outstanding performance in various applications. Particularly, MOFs have been gaining increasing attention as catalysts because their catalytic activities can be tuned relatively easily by controlling their compositional and structural characteristics. In most cases, the well-developed micropores of MOFs are a significant advantage for appropriate mass transport during catalytic reactions, and the open metal sites within a porous framework act as active sites for such catalytic reactions^28–30^. Nonetheless, most of the binding sites in MOFs are already coordinated by organic linkers as a part of the framework, and hence, it is difficult for the reactants to interact with these active metals. Therefore, the construction of well-ordered crystalline MOFs having many open metal sites, in which the reactants can easily interact with active metals, is an ideal direction for the preparation of highly active MOF-based catalysts^31–35^.

Herein, we report a strategy for enhancing the catalytic activity of MOF-74 by conferring additional open metal sites within its structure. The additional open metal sites were generated by replacing some of the original organic linkers (2,5-dihydroxy-1,4-bezenedicarboxylic acid, DHBDC) incorporated within a MOF-74 by analogous organic linkers (2-hydroxy-1,4-benzenedicarboxylic acid, HBDC), that resulted in the structural defects (excluding some of the bridged hydroxyl groups) within the MOF-74 structure and consequently led to the generation of additional active metal sites. The resulting MOF-74 displayed the enhanced catalytic activity for the cyanosilylation of several aldehydes owing to the additional open metal sites generated within the MOF-74 structure. Moreover, the chemical composition of MOF-74 was tuned by altering the blending ratios of the two organic linkers, and the optimum blending ratio for the best catalytic performance was determined.

## Results and discussion

**Figure 1:**
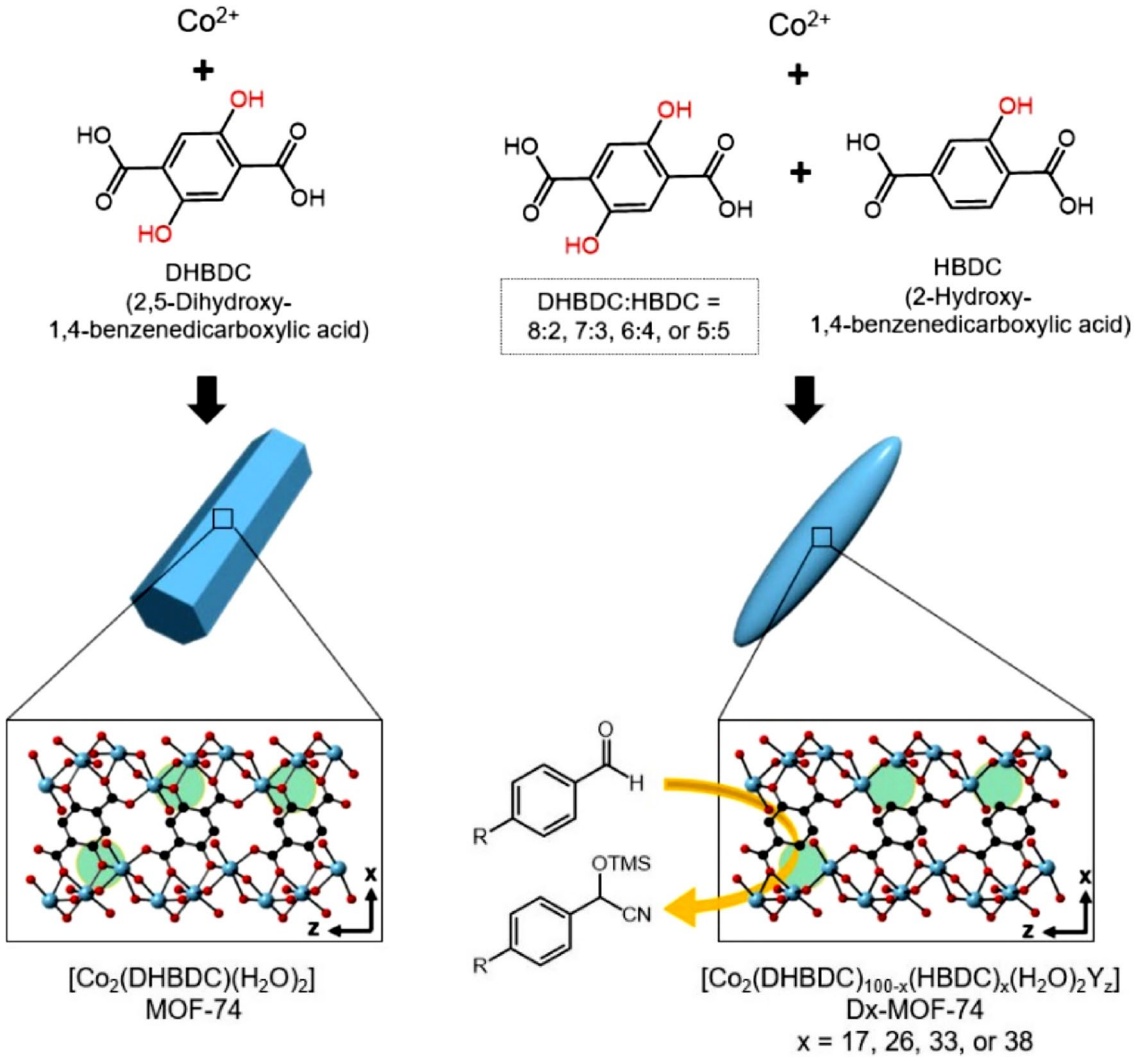
Schematic of the preparation of MOF-74 from Co^2+^ and DHBDC (left) and the preparation of Dx-MOF-74 from Co^2+^ and several ratios of the two organic linkers (DHBDC:HBDC = 8:2, 7:3, 6:4, or 5:5) as heterogeneous catalysts for the catalytic cyanosilylation of aldehydes. The x in Dx-MOF-74 represents the incorporated percent of HBDC.

**Figure 2:**
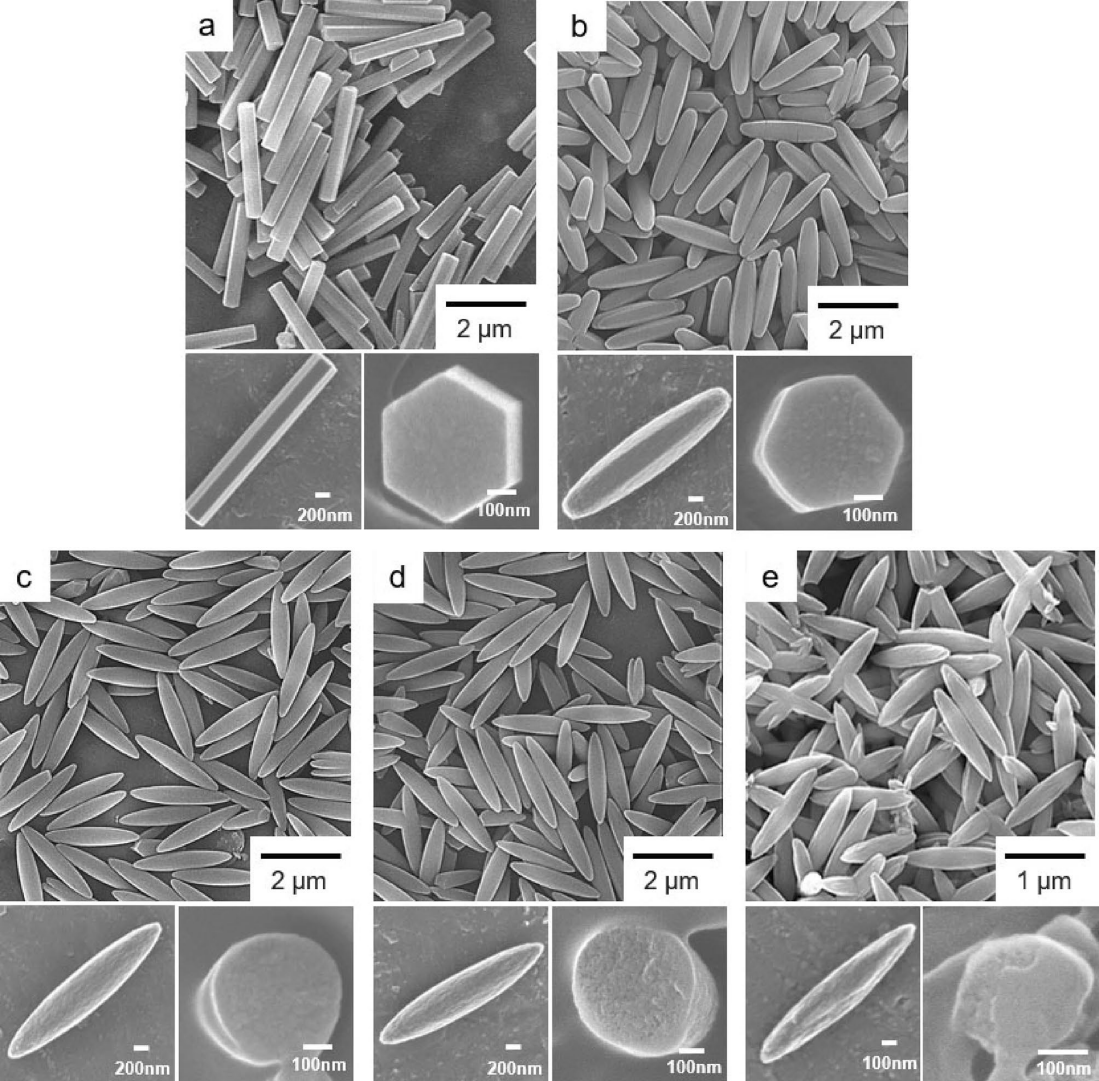
SEM images showing the morphological change from (**a**) hexagonal rods of pure MOF-74 to (**b**) truncated hexagonal rods of D17-MOF-74 and (**c**–**e**) rice-shaped particles of D26-MOF-74, D33-MOF-74, and D38-MOF-74, respectively. A series of D-MOF-74 samples was prepared from Co^2+^ and several ratios of DHBDC and HBDC, (**b**) 8:2 (D17-MOF-74), (**c**) 7:3 (D26-MOF-74), (**d**) 6:4 (D33-MOF-74), and (**e**) 5:5 (D38-MOF-74). Enlarged SEM images and SEM images showing the cross-sections of the particles are displayed in the insets.

**Figure 3:**
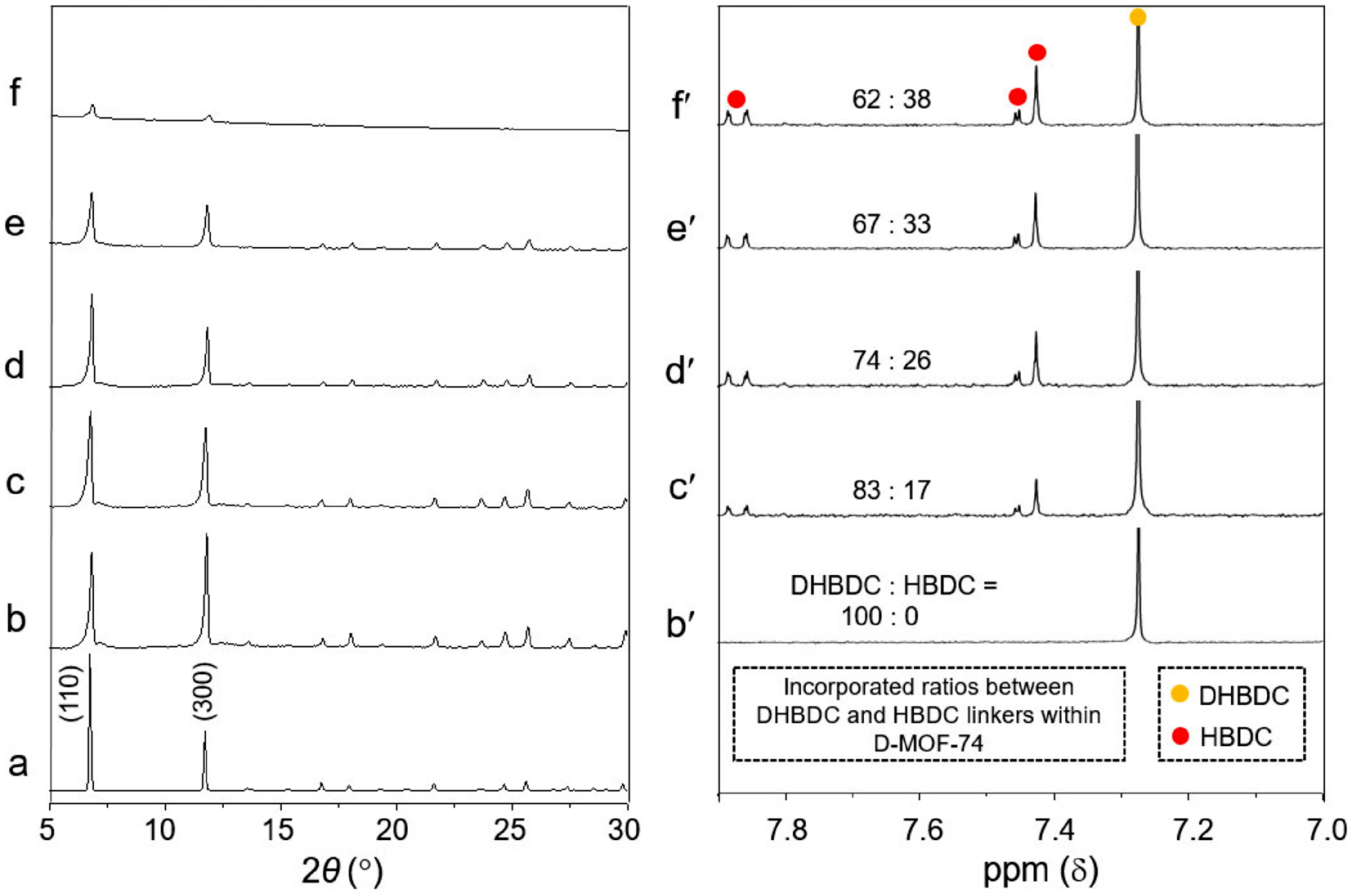
(**a**) Simulated PXRD pattern of MOF-74. PXRD patterns of the synthesized, (**b**) MOF-74, (**c**) D17-MOF-74, (**d**) D26-MOF-74, (**e**) D33-MOF-74, and (**f**) D38-MOF-74. Corresponding ^1^H NMR spectra of (**b′**) MOF-74, (**c′**) D17-MOF-74, (**d′**) D26-MOF-74, (**e′**) D33-MOF-74, and (**f′**) D38-MOF-74.

First, MOF-74, with a chemical composition of [Co_2_(DHBDC)(H_2_O)_2_]_*n*_ and a 3D hexagonal structure, was synthesized via the solvothermal reaction of CoCl_2_ and DHBDC^36^ (Fig. 1). A cobalt version of MOF-74 having uniform morphology and size can be made via this solvothermal reaction. And the morphological purity of product is critical in determining the purity of product. Morphological and structural characteristics of the resulting product were examined by scanning electron microscopy (SEM) and powder X-ray diffraction (PXRD). SEM images of the product revealed the formation of uniform micro-sized hexagonal rods (Fig. 2a). In particular, the hexagonal facet of the resulting rods is representative of the hexagonal structure of MOF-74 (inset in Fig. 2a). In addition, the PXRD pattern of the product (Fig. 3b), which was identical to the pattern simulated from the single-crystal X-ray structure of MOF-74^37,38^ (Fig. 3a), confirmed the formation of MOF-74. Following this, a series of MOF-74 samples with structural defects (denoted as D-MOF-74) were prepared through similar solvothermal reactions of CoCl_2_ and DHBDC but in the presence of an analogous organic linker (HBDC), as shown in Fig. 1. The ratio of the two organic linkers used was maintained at 8:2, 7:3, 6:4, or 5:5 (DHBDC: HBDC). By the way, the similar solvothermal reaction of CoCl_2_ and pure HBDC resulted in the amorphous material instead of a crystalline MOF-74 analogue (Fig. S1). As illustrated in Fig. 1, the replacement of some of the DHBDC linkers by HBDC resulted in the structural defects (excluding some of the bridged hydroxyl groups) with extra open metal sites within the framework. Typically, MOF-74 is constructed through the coordination of Co^2+^ with the carboxyl groups and hydroxyl groups of DHBDC and consists of the CoO_6_ octahedra^37,38^. In the CoO_6_ octahedra, three oxygen atoms come from the carboxyl groups and two oxygen atoms come from the hydroxyl groups as a bridge mode (Fig. 1 and S2). The last oxygen atom in the CoO_6_ octahedra is that of a water molecule, and this site can act as an open metal site during the catalytic reactions, such as cyanosilylation of aldehyde, oxygen evolution reaction, NO_x_ reduction, and cyclohexane oxidation^30,32,39,40^. When some of the DHBDC linkers within a framework are replaced by HBDC, additional open metal sites are generated owing to the disappearance of some of the coordination bonds between Co^2+^ and a bridged hydroxyl group (see green circles in Fig. 1). The open metal sites generated due to the missing bridged hydroxyl groups are exposed toward the hexagonal channels of MOF-74, as shown in Fig. S3. And thus, the reactants during the catalytic reaction can interact with these open metal sites through the hexagonal channels. The additional anions should be involved in the framework for the charge valance due to the incorporation of HBDC instead of DHBDC. Indeed, X-ray photoelectron spectroscopy (XPS) profiles of D-MOF-74 samples revealed the presence of chloride ions as counteranions (Fig. S4). The energy dispersive X-ray (EDX) spectra also exhibited the presence of Cl atom (Fig. S5). In addition to chloride ions, extra neutral molecules, such as water and solvent molecule, should be coordinated to cobalt ions in place of the missing bridged hydroxyl groups. In a series of D-MOF-74, the number of open metal sites should increase with increasing amount of HBDC. Eventually, these additional open metal sites should be beneficial for enhancing the catalytic activity of MOF-74.

The chemical compositions of a series of D-MOF-74 were analyzed by EDX spectroscopy. Peaks corresponding to Co, C, O and Cl were detected in the EDX spectra, thereby confirming the presence of cobalt ions, organic linkers, and chloride ions (Fig. S5). The actual blending ratio of DHBDC and HBDC in D-MOF-74 was determined by ^1^H NMR spectroscopy (Fig. 3). First, the series of D-MOF-74 were digested in a mixed deuterated-solvent of DCl and DMSO-d_6_ to measure NMR spectra. And the peak integrations were used to determine the relative amounts of the two organic linkers. The blending ratios of DHBDC and HBDC in the series of D-MOF-74 were found to be 83:17, 74:26, 67:33, and 62:38 (Fig. 3c′–f′). Henceforth, the D-MOF-74 samples will be denoted by the amount of HBDC incorporated within the framework; for example, D17-MOF-74 refers to MOF-74 incorporated with 17% HBDC. The theoretical open metal sites of a series of D-MOF-74 were calculated to be increased ca. 34, 52, 66, and 76% for D17-MOF-74, D26-MOF-74, D33-MOF-74, and D-38-MOF-74, respectively, due to the incorporation of HBDC compared to that of pure MOF-74.

Morphological change in the D-MOF-74 samples was detected in SEM images. SEM images revealed the formation of uniform and well-defined rods with a high aspect ratio, similar to that of pure MOF-74 (Fig. 2). First, the morphological homogeneity of samples (Fig. 2) indicated the formation of one kind product instead of the mixture of a crystalline MOF-74 from Co^2+^ and DHBDC and an amorphous product from Co^2+^ and HBDC. If the mixed products were generated from the reactions, the mixture of hexagonal rods of pure MOF-74 and wire-type materials should be observed in SEM images instead of a uniform rice-shaped particle. As mentioned earlier, the solvothermal reaction of CoCl_2_ and HBDC resulted in the amorphous product with a wire shape instead of a well-defined hexagonal rods of a crystalline MOF-74 (Fig. S1). The characteristic hexagonal shape with angled edges observed in pure MOF-74 (Fig. 2a) transformed to rounded edges in D-MOF-74 (Fig. 2b-e). The magnified SEM images clearly displayed the morphological change from the angled edges to the rounded edges. Although the hexagonal shape was retained to some extent in D17-MOF-74 (Fig. 2b), the hexagonal shape with angled edges completely disappeared with increasing amount of the HBDC linkers in the framework and increasing structural defects (Fig. 2c-e). SEM images of the sliced samples, which are prepared by embedding samples in an epoxy resin and slicing to expose the cross-section, clearly displayed their characteristic cross-sections (insets in Fig. 2). Truncated hexagonal rods were observed in D17-MOF-74, while rice-shaped microparticles were observed in other D-MOF-74 samples. Typically, the rounded feature of the particle is representative of its amorphous characteristic^41,42^. In specific, the morphological change of MOF particles from the angled edges to the rounded edges was found to be related to the crystallinity change from the crystalline to the amorphous materials^42^. Structural defects (excluding some of the bridged hydroxyl groups) impart an amorphous characteristic to the crystalline materials through the disorder of some of the well-ordered structural patterns of the crystalline materials. Nevertheless, the PXRD patterns of the D-MOF-74 samples revealed that all these samples technically had a 3D hexagonal structure of MOF-74 (Fig. 3c–f). However, the peak intensities were found to decrease in the PXRD patterns of D-MOF-74 (PXRD measurements of samples were conducted using an identical amount). Particularly, D38-MOF-74 exhibited very weak signals for the (110) and (300) planes owing to the increase in the amorphous characteristic.

**Figure 4:**
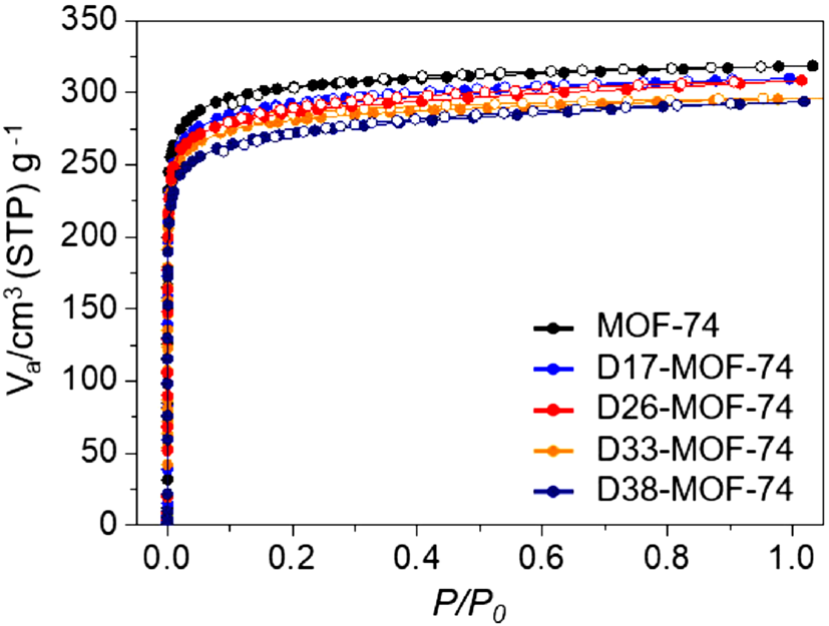
N_2_ sorption isotherms of pure MOF-74 and a series of D-MOF-74 samples.

Porosities of a series of D-MOF-74 samples were determined from their N_2_ sorption isotherms. With increasing amount of HBDC, the BET surface areas and total pore volumes of D-MOF-74 decreased slightly compared to those of pure MOF-74 (Fig. 4). This is due to some of the amorphous features of D-MOF-74 originated from the incorporation of the HBDC linkers. The BET surface areas and total pore volumes of pure MOF-74 were 1182.8 m^2^ g^-1^ and 0.49 cm^3^ g^-1^, respectively. However, the BET surface areas of the D-MOF-74 samples were 1140.4, 1119.3, 1105.7 and 1053.2 m^2^ g^-1^, while the total pore volumes were 0.48, 0.48, 0.46, and 0.45 cm^3^ g^-1^ (Table S1). The pore size distributions^43–45^ of the D-MOF-74 samples were calculated using the non-local density functional theory; no significant difference compared to that of pure MOF-74 was observed (Fig. S6). The thermogravimetric analysis (TGA) curves of samples were also measured to check their thermal stabilities (Fig. S7); however, there is no significant difference among the samples.

Cyanosilylation is an important route for the synthesis of cyanohydrins from aldehydes, which are vital compounds for the production of several fine chemicals including pharmaceuticals^46–49^. The enhanced Lewis acid catalytic activity of MOF-74 through providing the additional open metal sites was demonstrated by conducting the cyanosilylation of several aldehydes. In general, the enhanced catalytic activity of D-MOF-74 is expected with increasing number of open metal sites within the framework resulting from the incorporation of the HBDC linkers. First, benzaldehyde was reacted with trimethylsilyl cyanide (TMSCN) under solvent-free conditions at 60 °C for 30 min in the presence of a catalytic amount of MOF-74 or D-MOF-74 (Table 1). The conversion of benzaldehyde to 2-phenyl-2-[(trimethylsilyl)oxy] acetonitrile was monitored by ^1^H NMR spectroscopy (Fig. S8). In addition, ^13^C NMR and mass spectroscopy measurements were conducted to confirm the formation of 2-phenyl-2-[(trimethylsilyl)oxy] acetonitrile (see experimental section). Overall, the conversion rates in the presence of the D-MOF-74 samples were higher than that in the presence of pure MOF-74 (Table 1). The conversion of benzaldehyde in the presence of pure MOF-74 was 67% in 30 min, while it increased to 73% and 93% in the presence of D17-MOF-74 and D26-MOF-74, respectively (Table 1). The conversions in the presence of D33-MOF-74 and D38-MOF-74 were higher (80 and 79%, respectively) than that in the presence of pure MOF-74 (67%) but were lower than that in the presence of D26-MOF-74 (93%). The lower catalytic activities of D33-MOF-74 and D38-MOF-74 compared to D26-MOF-74 despite an increase in the amount of HBDC can be attributed to excess structural defects. The catalytic activity of D-MOF-74 should be affected not only by the number of open metal sites but also by the structural features. Excess structural defects due to the incorporation of too much HBDC results in the disorder of some of the well-ordered framework, thereby hindering the smooth chemical transportation during the catalytic reaction. Considering all these factors, D26-MOF-74 was found to be the best among the D-MOF-74 samples. In addition, the cyanosilylation of three other aldehydes (4-methoxybenzaldehyde, 4-methylbenzaldehyde, and 4-fluorobenzaldehyde) was conducted in the presence of a catalytic amount of D26-MOF-74 or MOF-74 to confirm the enhanced catalytic activity of D26-MOF-74 (Fig. S9, S10, and S11). As expected, the conversions of all the three aldehydes were higher in the presence of D26-MOF-74 than in the presence of pure MOF-74 (Table 2). The fast cyanosilylation of aldehydes bearing electron donating groups is previously reported compared to that of aldehydes bearing electron withdrawing groups^50,51^.

**Table 1:** Cyanosilylation^*a*^ of benzaldehyde with TMSCN catalyzed by MOF-74 or a series of D-MOF-74.


Entry	Catalyst	Yield (%)
1	MOF-74	67
2	D17-MOF-74	73
3	D26-MOF-74	93
4	D33-MOF-74	80
5	D38-MOF-74	79

^*a*^Reaction conditions: benzaldehyde (1 mmol), catalyst (1.7 mol% based on Co(II)), trimethylsilyl cyanide (TMSCN; 3 mmol). No solvent was added. Conversion was monitored by ^1^H NMR spectroscopy after 30 min of reaction.

**Table 2:** Cyanosilylation^*a*^ of various aldehydes with TMSCN catalyzed by MOF-74 or D26-MOF-74.


Entry	Substrate	Yield (%)	
		MOF-74	D26-MOF-74
1		67	93
2^*b*^		58	83
3		71	95
4		50	72

^*a*^Reaction conditions: aldehyde (1 mmol), catalyst (1.7 mol% based on Co (II)), trimethylsilyl cyanide (TMSCN; 3 mmol). No solvent was added. Conversion was monitored by ^1^H NMR spectroscopy after 30 min of reaction.

^*b*^Reaction time is reduced to 10 min due to the fast reaction rate.

**Figure 5:**
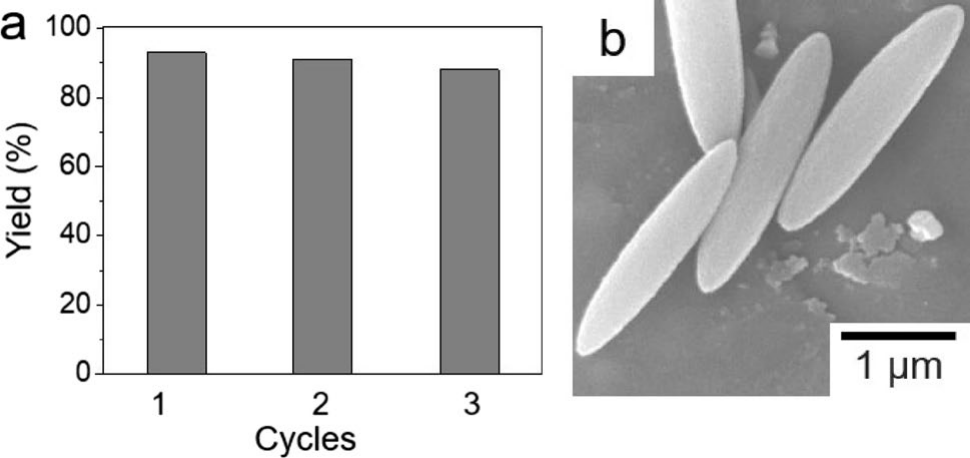
(**a**) Recyclability of D26-MOF-74 over three catalytic cycles. (**b**) SEM image of D26-MOF-74 after three catalytic cycles.

The recyclability of D26-MOF-74 was also tested. The conversion rate of benzaldehyde was well maintained even after three catalytic cycles, as shown in Fig. 5a. In addition, SEM image of D26-MOF-74 after three successive catalytic cycles revealed no morphological change from long rice-shape rods (Fig. 5b), and the PXRD patterns of D26-MOF-74 measured before and after three catalytic cycles displayed no critical structural change (Fig. S12).

## Conclusion

In summary, a series of well-defined and uniform rice-shaped MOF-74 microparticles with additional open metal sites were prepared by blending two analogous organic linkers (DHBDC and HBDC). The replacement of some of the DHBDC linkers within the MOF-74 structure with HBDC linkers resulted in the structural defects, with additional open metal sites within the MOF-74 structure, owing to the disappearance of some of the bridged hydroxyl groups. These extra open metal sites were found to be beneficial for the catalytic cyanosilylation of several aldehydes. In addition, the optimum blending ratio of DHBDC and HBDC within MOF-74 to achieve the best catalytic performance was determined, and the excellent recyclability of the defected MOF-74 for the catalytic reaction was demonstrated.

## Experimental

### General methods

All chemicals and solvents were purchased from commercial sources and used as received. All scanning electron microscopy (SEM) images were obtained using a JEOL IT-500HR field-emission SEM and Carl Zeiss SUPRA 55VP field-emission SEM (National Instrumentation Centre for Environmental Management, Seoul National University). For the investigation of the cross-section of the particles, the particles were embedded in a mixture of epoxy resin and the placed in an oven at 70 °C for 8 h. The cross-section of the particles was obtained using HS-1 Vertical Slicer. Powder X-ray diffraction (PXRD) patterns were acquired using a Rigaku Ultima IV equipped with a graphite monochromated Cu Kα radiation source (40 kV, 40 mA). X-ray photoelectron spectroscopy (XPS) measurements were carried out on a Thermo Scientific K-Alpha KA1066 spectrometer using a monochromatic Al Kα X-ray source (hν = 1486.6 eV). Energy dispersive X-ray spectroscopy (EDX) spectra were acquired using a Hitachi SU 1510 SEM with a Horiba EMAX Energy E-250 EDS system. Thermogravimetric analysis (TGA) curves were obtained using a Shimadzu TGA-50 under a nitrogen atmosphere with heating rate of 5 °C min^−1^. N_2_ sorption isotherms (77 K) were measured using a BELSORP Max volumetric adsorption system. All sorption isotherms were measured after pretreatment under a dynamic vacuum at 120 °C for 12 h. ^1^H and ^13^C NMR spectra were recorded on a Bruker Avance III HD 300 spectrometer (^1^H NMR, 300 MHz) (^13^C NMR, 75 MHz) with chemical shifts reported relative to residual deuterated solvent peaks. GC-MS data was acquired using an Agilent 7890B/5977A instrument.

### Preparation of MOF-74^36^

CoCl_2_·6H_2_O (0.30 mmol, 71.4 mg), 2,5-dihydroxy-1,4-benzenedicarboxylic acid (DHBDC) (0.14 mmol, 27.1 mg), and polyvinylpyrrolidone (PVP) (250 mg) were dissolved in 5 mL of N,N-dimethylformamide (DMF). The mixture was placed in an oil bath at 120 °C for 2 h. The product was isolated via centrifugation, washed with DMF and methanol, and dried in vacuum for 1 h. Among the several synthetic methods for the production of MOF-74, this solvothermal method^36^ was adapted for the production of MOF-74 having uniform morphology and size.

### Preparation of a series of D-MOF-74

CoCl_2_·6H_2_O (0.30 mmol, 71.4 mg), DHBDC (0.11, 0.10, 0.08, or 0.07 mmol), 2-hydroxy-1,4-benzenedicarboxylic acid (HBDC) (0.03, 0.04, 0.06, or 0.07 mmol), and PVP (250 mg) were dissolved in 5 mL of DMF. The mixture was placed in an oil bath at 120 °C for 2 h. The product was isolated via centrifugation, washed with DMF and methanol, and dried in vacuum for 1 h.

### Cyanosilylation of aldehydes with trimethylsilyl cyanide

Samples (MOF-74 and D-MOF-74 catalysts) were used for the catalytic reactions after a simple dry process without a harsh evacuation process^52,53^. Aldehyde (1 mmol) and trimethylsilyl cyanide (TMSCN) (3 mmol) were added into catalyst (3.0 mg) in a 4 mL teflon vial. The suspension was stirred at 60 °C for 30 min. After 30 min, the catalyst was isolated via centrifugation. ^1^H NMR spectra were recorded to determine the activity of the catalyst. In the recycling test, total third cycles of the cyanosilylation of benzaldehyde were repeated with D26-MOF-74 catalyst. After first catalytic reaction, catalyst was isolated via centrifugation, washed with methanol several times, and dried under vacuum. Then fresh benzaldehyde and TMSCN were added into the vial containing the used catalyst for second cycle catalytic reaction. The same procedure was repeated for third cycle catalytic reaction. 2-phenyl-2-((trimethylsilyl)oxy)acetonitrile. ^13^C NMR (MHz, CDCl_3_): δ 136.42, 129.44, 129.04, 126.45, 119.29, 63.74, − 0.17. GC-MS [M+H]^+^ = 206.1. 2-(4-methoxyphenyl)-2-((trimethylsilyl)oxy)acetonitrile. ^13^C NMR (MHz, CDCl_3_): δ 160.47, 128.61, 128.04, 119.46, 114.38, 63.44, 55.43, − 0.14. GC-MS [M]^+^ = 235.2. 2-(p-tolyl)-2-((trimethylsilyl)oxy)acetonitrile. ^13^C NMR (MHz, CDCl_3_): δ 139.43, 133.56, 129.69, 126.50, 119.42, 63.66, 21.28, − 0.14. GC-MS [M+H]^+^ = 220.1. 2-(4-fluorophenyl)-2-((trimethylsilyl)oxy)acetonitrile. ^13^C NMR (MHz, CDCl_3_): δ 164.89, 132.42, 128.36, 119.12, 116.19, 63.07, − 0.21. GC-MS [M+H]^+^ = 224.0.

## Supplementary Information

Supplementary Information.

## Data Availability

All data generated or analysed during this study are included in this published article and its supplementary information files.
