# Supplementary material for: Enhanced catalytic activity of MOF-74 via providing additional open metal sites for cyanosilylation of aldehydes
Source: Sci Rep. 2022 Aug 30;12:14735. doi: 10.1038/s41598-022-18932-z (PMC9427751; doi:10.1038/s41598-022-18932-z)
Supplement: Supplementary file 1 — Supplementary Information. [file 41598_2022_18932_MOESM1_ESM.pdf]

Supplementary Information

Enhanced catalytic activity of MOF-74 via providing additional open metal sites for cyanosilylation of aldehydes

*Hyeji Jun, Sojin Oh, Gihyun Lee and Moonhyun Oh**

Department of Chemistry, Yonsei University, 50 Yonsei-ro, Seodaemun-gu, Seoul 03722, Republic of Korea
*Corresponding author.
E-mail: moh@yonsei.ac.kr

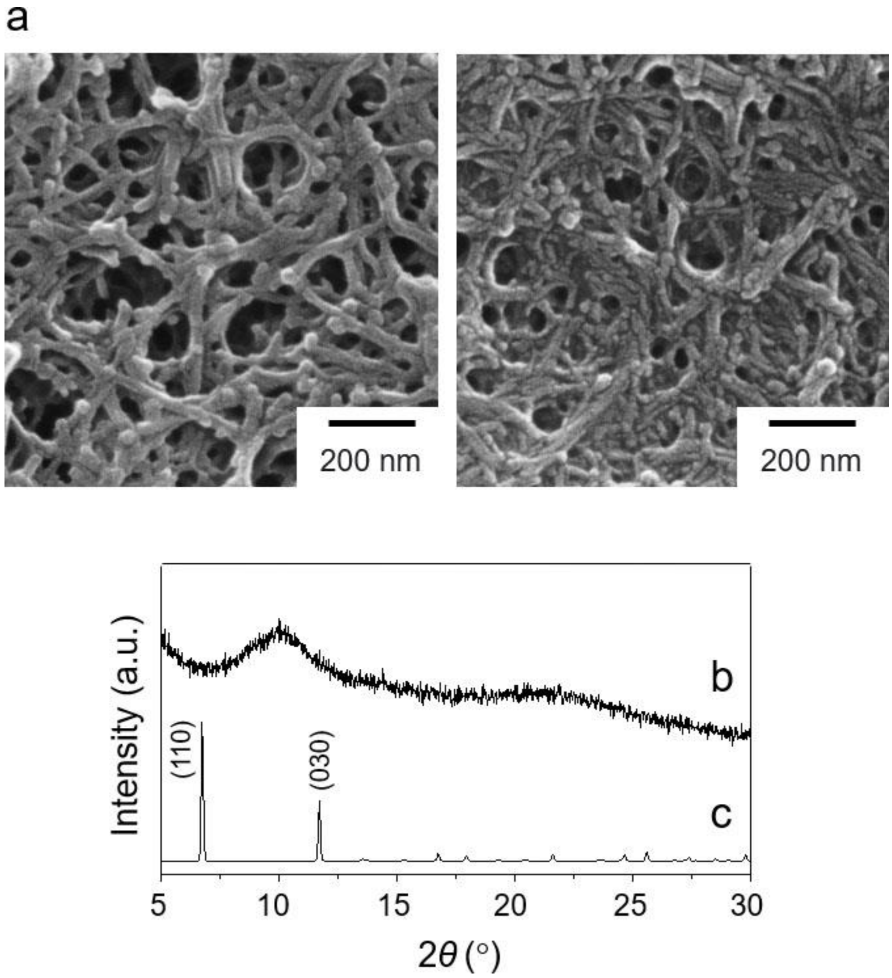

Figure S1. (a) SEM images and (b) PXRD pattern of the product obtained from the solvothermal reaction of $CoCl_2$ and HBDC instead of DHBDC. (c) Simulated PXRD pattern of MOF-74.

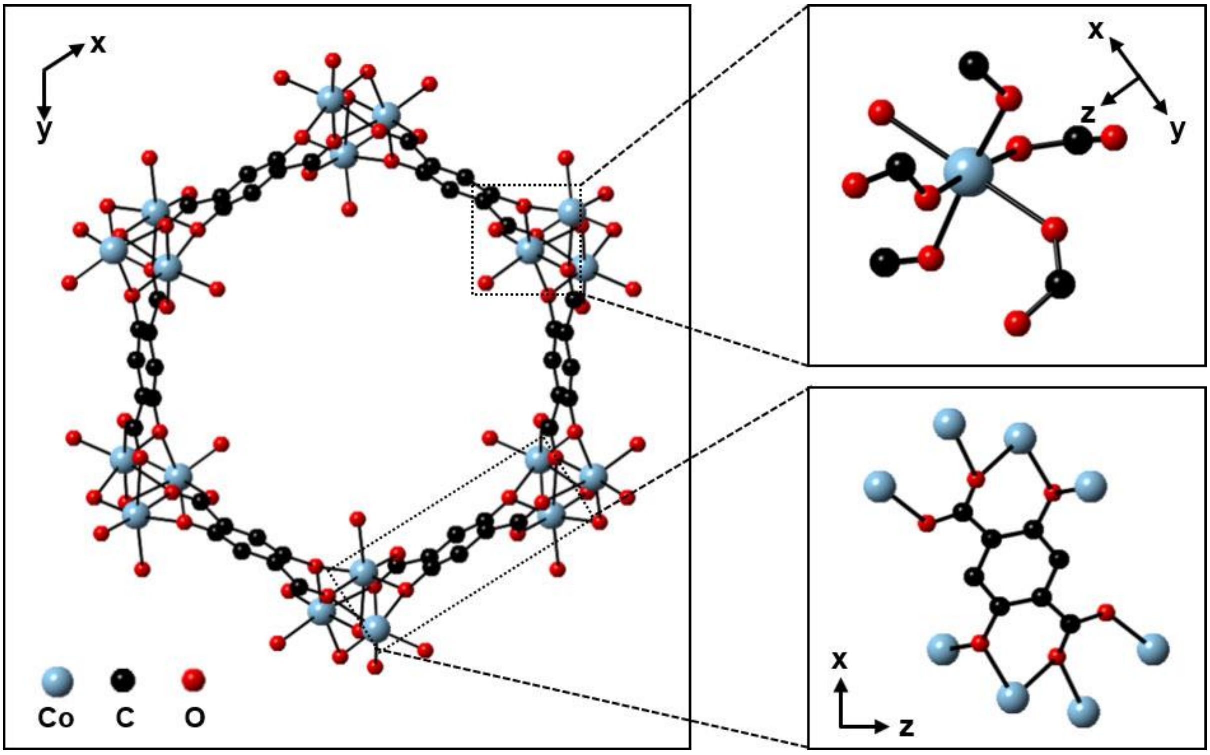

Figure S2. Ball-and-stick representations of MOF-74. Co: sky blue; C: black; O: red. H atoms are omitted for clarity.

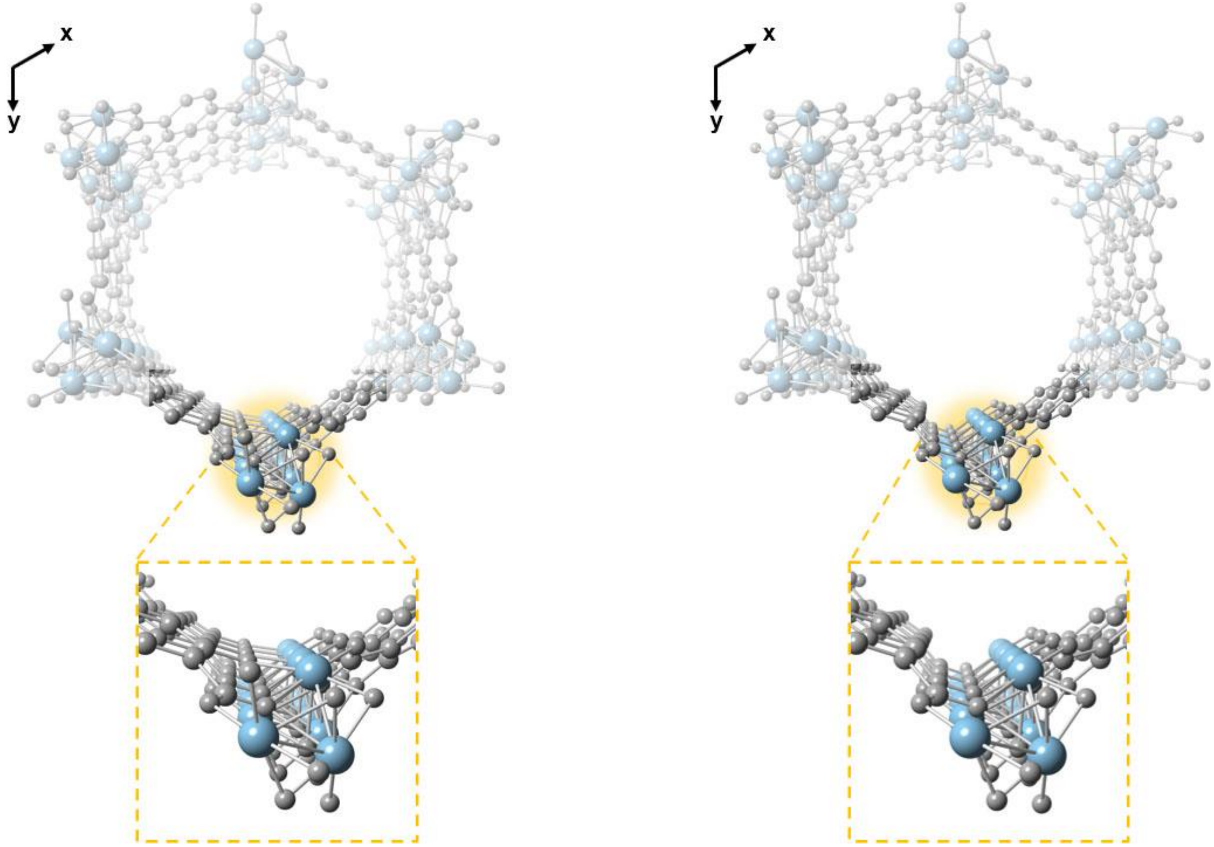

Figure S3. Ball-and-stick representations of MOF-74 (left) and D-MOF-74 (right) showing the open metal sites generated due to the missing bridged hydroxyl groups. The reactants can interact with these open metal sites through the hexagonal channels. Co: sky blue; C: grey; O: grey. H atoms and H_2O are omitted for clarity.

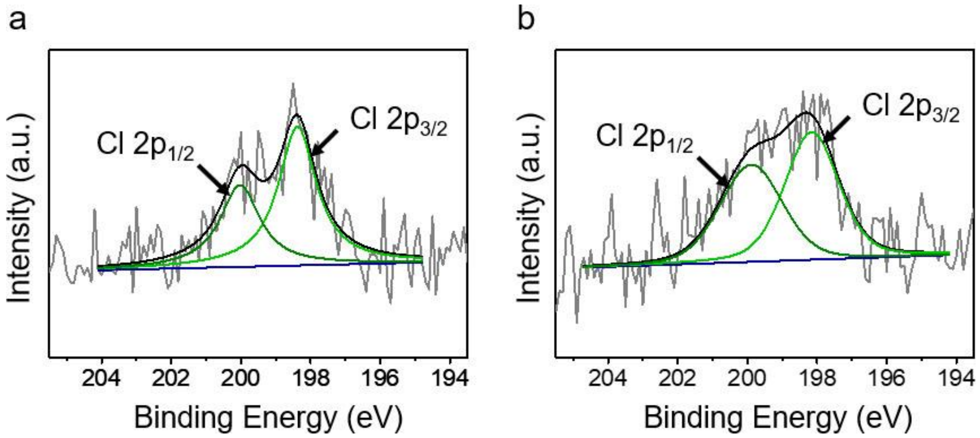

Figure S4. XPS spectra of (a) D26-MOF-74 and (b) D38-MOF-74.

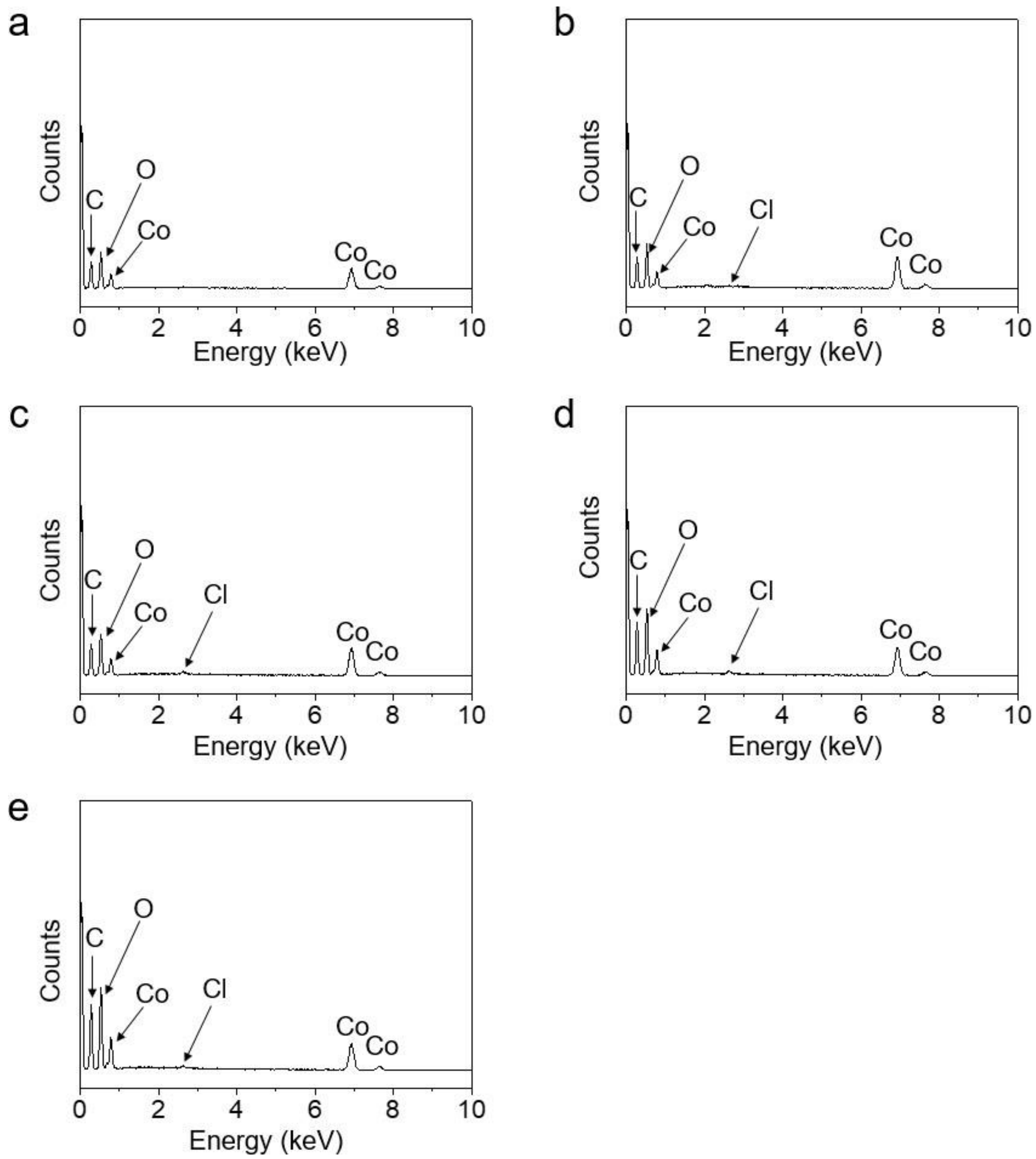

Figure S5. EDX spectra of (a) pure MOF-74, (b) D17-MOF-74, (c) D26-MOF-74, (d) D33-MOF-74, and (e) D38-MOF-74.

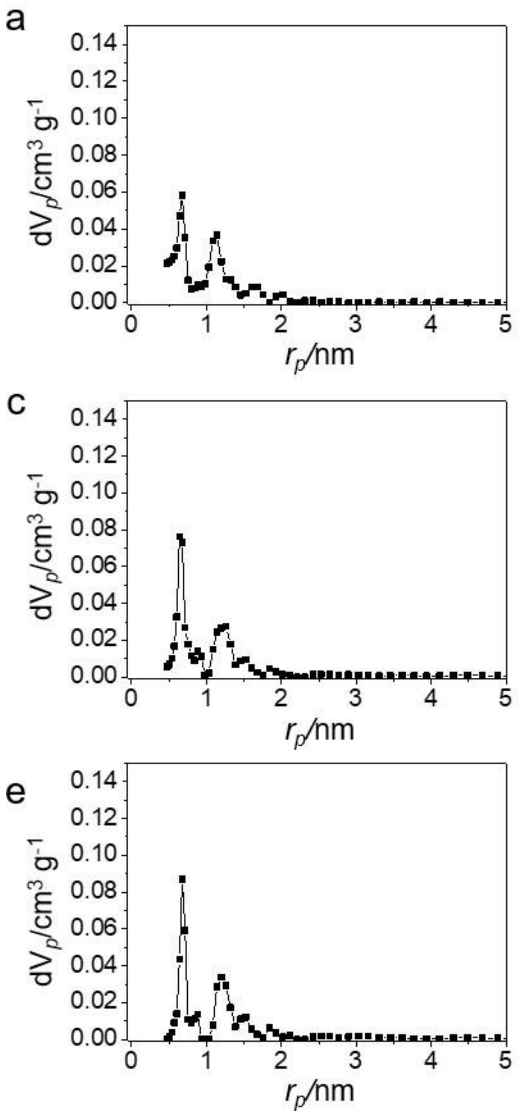

Figure S6. Pore size distributions of (a) pure MOF-74, (b) D17-MOF-74, (c) D26-MOF-74, (d) D33-MOF-74, and (e) D38-MOF-74 calculated using the non-local density functional theory (NLDFT).

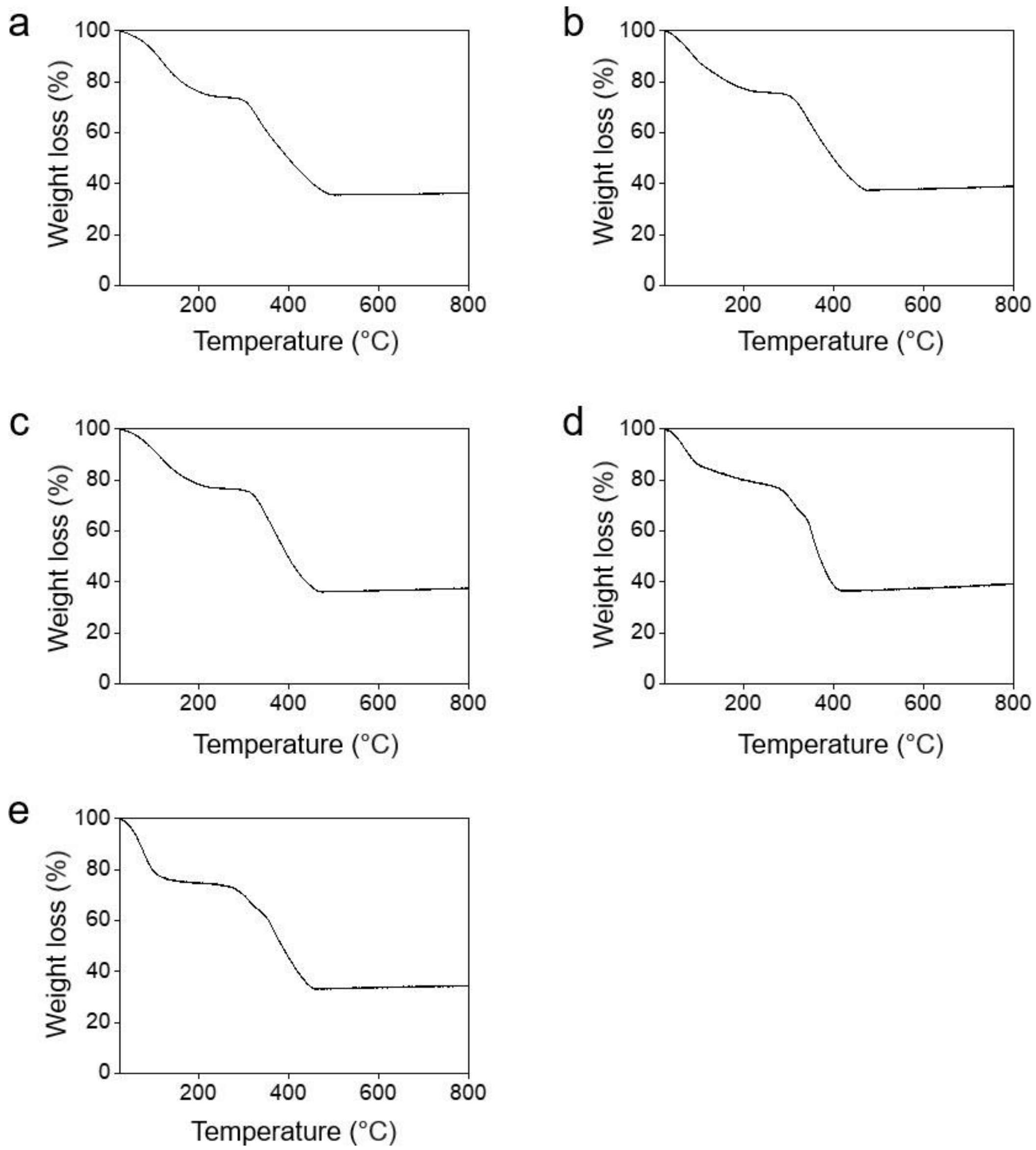

Figure S7. TGA curves of (a) pure MOF-74, (b) D17-MOF-74, (c) D26-MOF-74, (d) D33-MOF-74, and (e) D38-MOF-74. There is no significant difference among the samples.

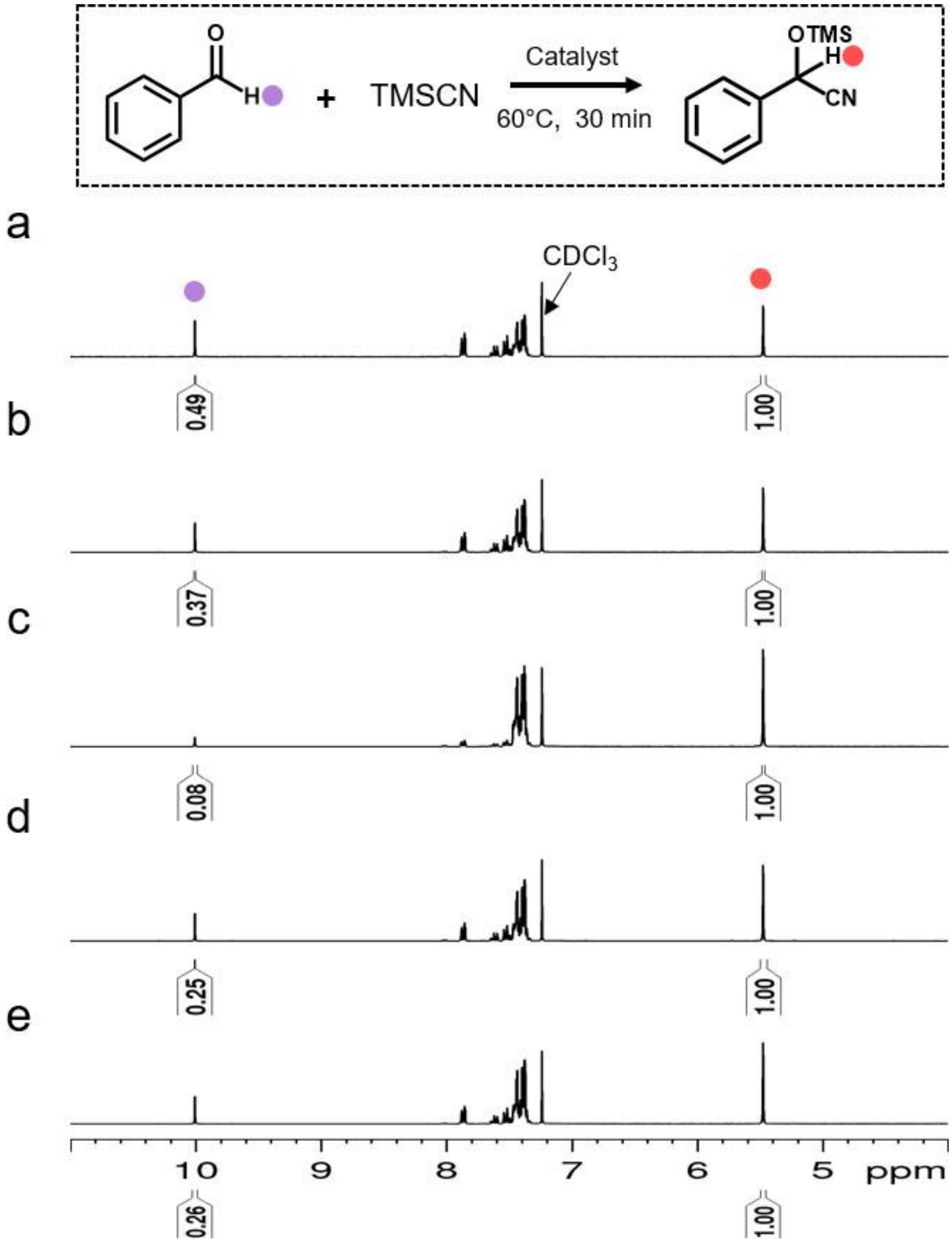

Figure S8. ^{1}H NMR spectra showing the conversion of benzaldehyde to 2-phenyl-2-[(trimethylsilyl)oxy] acetonitrile in the presence of a catalytic amount of (a) pure MOF-74, (b) D17-MOF-74, (c) D26-MOF-74, (d) D33-MOF-74, and (e) D38-MOF-74 after 30 min of reaction.

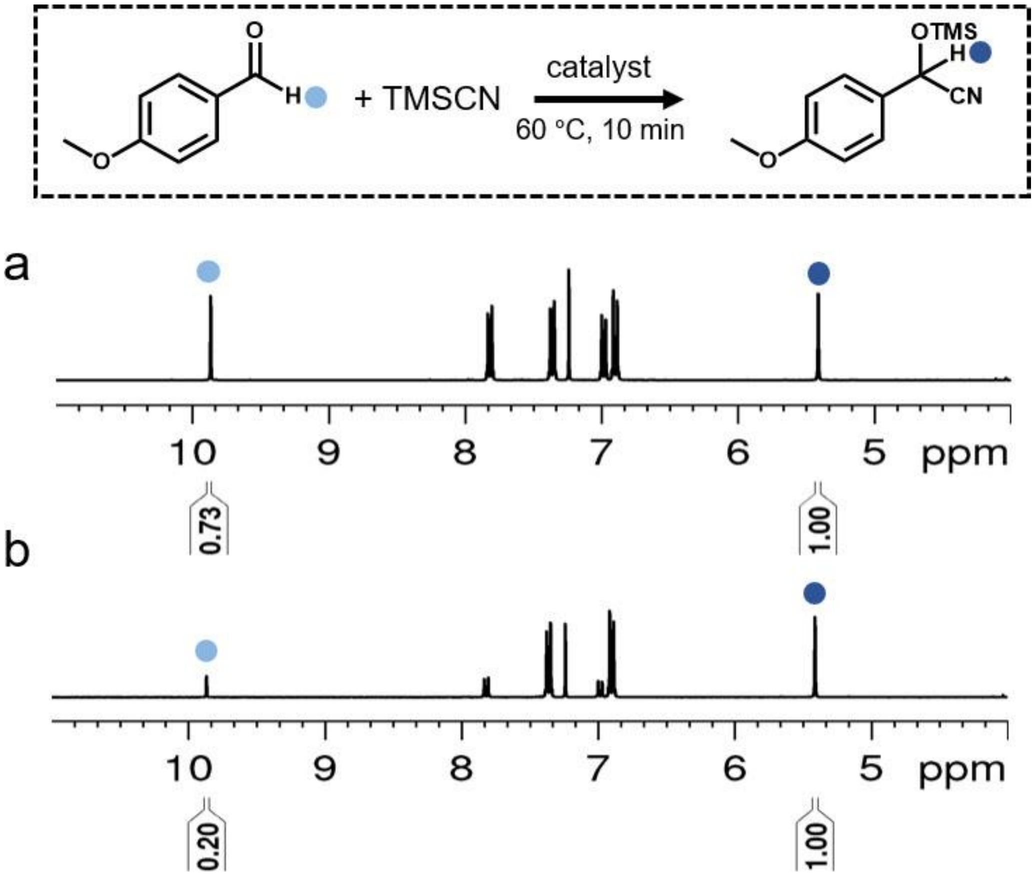

Figure S9. ^{1}H NMR spectra showing the conversion of 4-methoxybenzaldehyde in the presence of a catalytic amount of (a) pure MOF-74 and (b) D26-MOF-74 after 10 min of reaction.

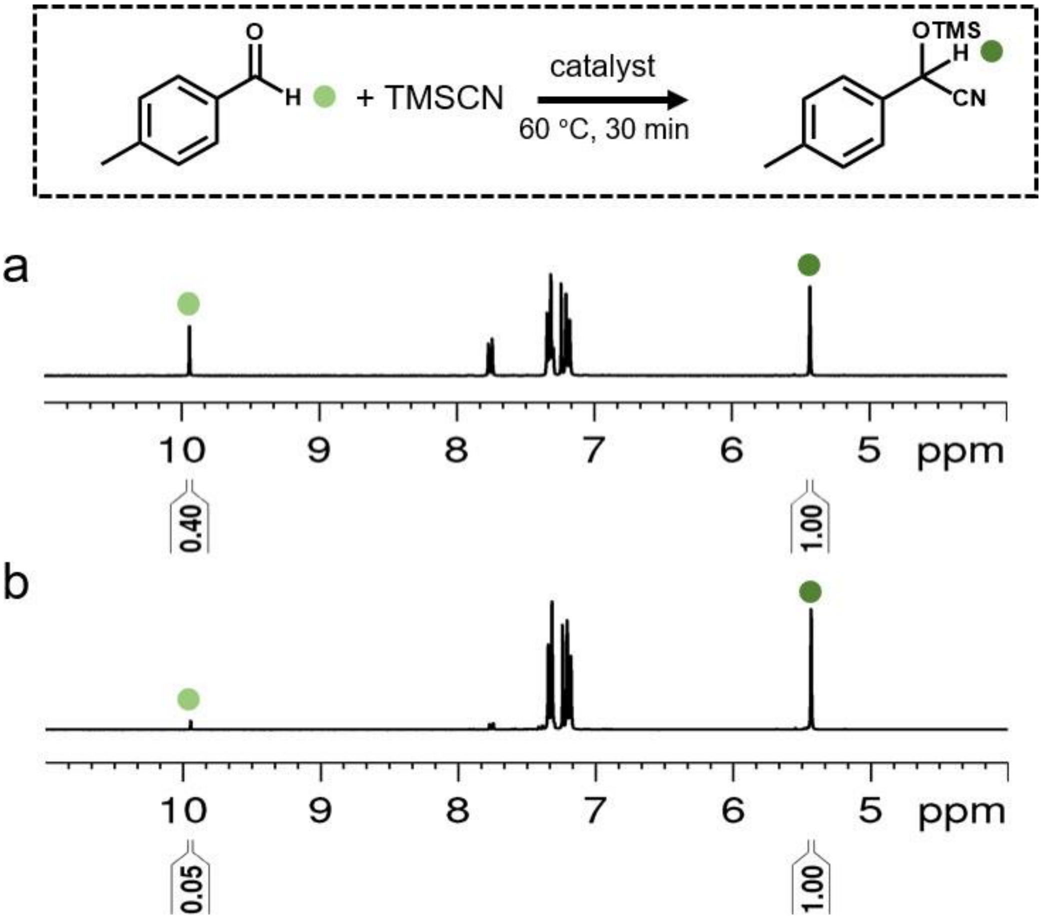

Figure S10. ^{1}H NMR spectra showing the conversion of 4-methylbenzaldehyde in the presence of a catalytic amount of (a) pure MOF-74 and (b) D26-MOF-74 after 30 min of reaction.

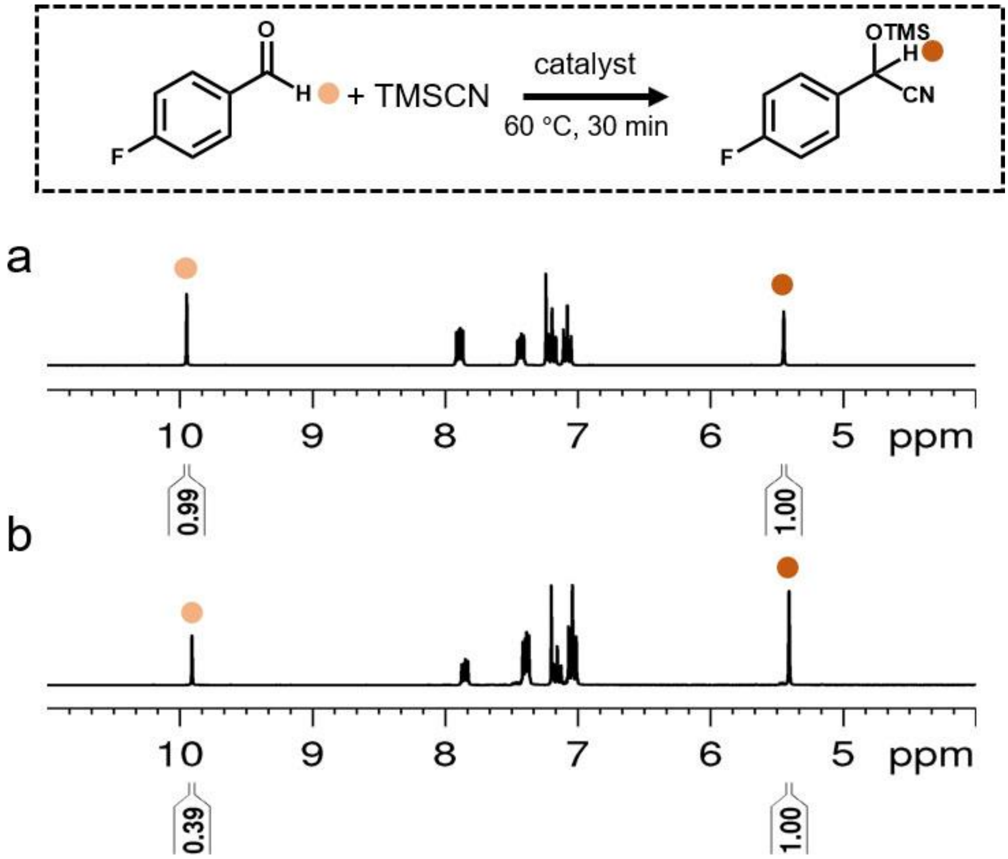

Figure S11. ^{1}H NMR spectra showing the conversion of 4-fluorobenzaldehyde in the presence of a catalytic amount of (a) pure MOF-74 and (b) D26-MOF-74 after 30 min of reaction.

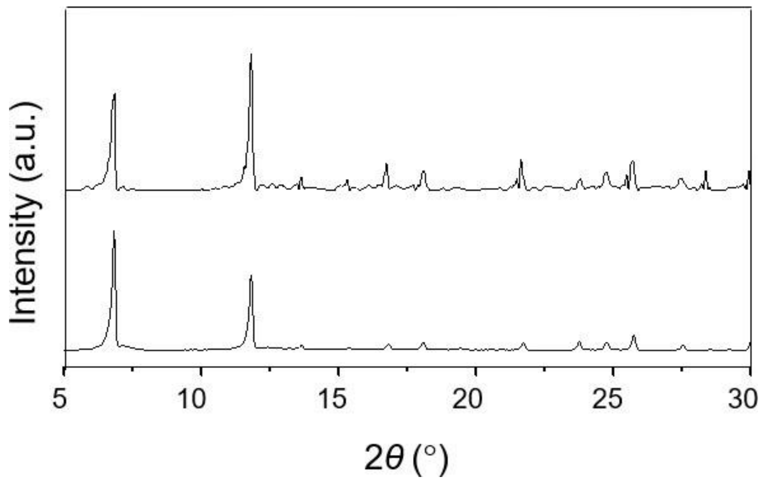

Figure S12. PXRD patterns of D26-MOF-74 measured before (bottom) and after (top) three catalytic cycles.

Table S1. BET surface areas and total pore volumes of pure MOF-74 and a series of D-MOF-74 samples.

	Surface area (m^2 g^{-1})	Total pore volume (cm^3 g^{-1})
MOF-74	1182.8	0.49
D17-MOF-74	1140.4	0.48
D26-MOF-74	1119.3	0.48
D33-MOF-74	1105.7	0.46
D38-MOF-74	1053.2	0.45
